# Automatic CT Angiography Lesion Segmentation Compared to CT Perfusion in Ischemic Stroke Detection: a Feasibility Study

**DOI:** 10.1007/s10278-022-00611-0

**Published:** 2022-02-24

**Authors:** Teemu Mäkelä, Olli Öman, Lasse Hokkinen, Ulla Wilppu, Eero Salli, Sauli Savolainen, Marko Kangasniemi

**Affiliations:** 1 HUS Medical Imaging Center, Radiology, University of Helsinki and Helsinki University Hospital, Haartmaninkatu 4, P.O. Box 340, 00290 Helsinki, Finland; 2 Department of Physics, University of Helsinki, P.O. Box 64, 00014 Helsinki, Finland

**Keywords:** Convolutional neural networks, Computed tomography angiography, Machine learning, Stroke

## Abstract

In stroke imaging, CT angiography (CTA) is used for detecting arterial occlusions. These images could also provide information on the extent of ischemia. The study aim was to develop and evaluate a convolutional neural network (CNN)–based algorithm for detecting and segmenting acute ischemic lesions from CTA images of patients with suspected middle cerebral artery stroke. These results were compared to volumes reported by widely used CT perfusion–based RAPID software (IschemaView). A 42-layer-deep CNN was trained on 50 CTA volumes with manually delineated targets. The lower bound for predicted lesion size to reliably discern stroke from false positives was estimated. The severity of false positives and false negatives was reviewed visually to assess the clinical applicability and to further guide the method development. The CNN model corresponded to the manual segmentations with voxel-wise sensitivity 0.54 (95% confidence interval: 0.44–0.63), precision 0.69 (0.60–0.76), and Sørensen–Dice coefficient 0.61 (0.52–0.67). Stroke/nonstroke differentiation accuracy 0.88 (0.81–0.94) was achieved when only considering the predicted lesion size (i.e., regardless of location). By visual estimation, 46% of cases showed some false findings, such as CNN highlighting chronic periventricular white matter changes or beam hardening artifacts, but only in 9% the errors were severe, translating to 0.91 accuracy. The CNN model had a moderately strong correlation to RAPID-reported *T*_max_ > 10 s volumes (Pearson’s *r* = 0.76 (0.58–0.86)). The results suggest that detecting anterior circulation ischemic strokes from CTA using a CNN-based algorithm can be feasible when accompanied with physiological knowledge to rule out false positives.

## Introduction

Applying artificial intelligence in medical research has experienced an exponential growth in interest over the past decades [1]. With its surging role in computer vision, deep convolutional neural networks (CNNs) hold promise for aiding physicians achieve an accurate imaging diagnosis and through automation facilitate screening efforts [2]. The general principle behind CNNs is to learn, without feature engineering, complex mappings from a data organized in a grid pattern (e.g., a 2D or 3D image) to a desired output (e.g., a segmentation, detection, diagnosis, or prognosis). Deep CNNs have been demonstrated to be well suited for many tasks in radiology [3], including stroke imaging [4]. Stroke, of which 87% are ischemic, is one of the leading causes of death and long-term disability [5]. Urgent imaging by novel state of the art computed tomography (CT) scanners and well-timed treatment of acute ischemic stroke (AIS) will reduce the burden of disease [6]. AIS imaging is a prime candidate for machine learning where improvements in diagnostic accuracy could directly lead to an improved quality of acute patient care [7]. Among other machine learning techniques, deep CNNs have been applied to single- and multi-contrast anatomical magnetic resonance (MR), diffusion-weighted imaging, MR perfusion, non-contrast CT, CT perfusion (CTP), and CT angiography (CTA) images for ischemic and hemorrhagic stroke lesion segmentation and vessel occlusion detection and in predicting endovascular therapy outcome and tissue fate [8–16]. As shown in a preceding paper, a CNN model is feasible in detecting ischemic parenchymal regions associated with acute thrombosis of the middle cerebral artery (MCA) in CTA images [17]. In addition to the published studies, several commercial solutions have been made available based on similar methods [18].

In this study, a CTA-based, hemispheric asymmetry aware, 42-layer-deep CNN model was developed, and its performance was compared to a commercial CTP-based software RAPID (iSchemaView, Menlo Park, California, USA). RAPID is an automated system using deconvolution of tissue and arterial signals to provide separate volume estimates for the ischemic penumbra (based on the time until the residue function reaches its peak, *T*_max_) and the ischemic core (cerebral blood flow < 30% of that in normal tissue) [19]. The performance of RAPID has been evaluated in earlier studies by comparing RAPID CTP analysis to diffusion–perfusion mismatch and prediction of final infarct volume from MR images [20]. The proposed deep CNN model does not process any CTP or MR diffusion data, providing an alternative with higher resolution and smaller ionizing radiation dose compared to CTP. The advantage of CT-based methods in general is that they are faster and more widely available than MRI in the emergency setting. Moreover, CTA is routinely acquired to detect large vessel occlusions, and the ischemic regions are not typically evaluated from these images. The proposed method could complement perfusion analysis (e.g., in cases where the perfusion study is non-diagnostic) without changes to the existing imaging protocols.

The aims of this feasibility study were to (1) evaluate the proposed CNN model trained on a small data set and compare the ischemic stroke lesion estimates to RAPID-reported volumes, (2) review false positive and false negative findings to guide the future development of the method, (3) investigate suitability for small lesion detection by determining how the detected lesion (or false positive) volumes compared to the initial stroke diagnosis, and (4) fully automate the workflow.

## Materials and Methods

### Image Acquisition and Patient Cohort

A total of 150 patients with a suspected AIS of the MCA were retrospectively selected for this study. Inclusion criteria were that the CT study needed to have a diagnostic CTA volume. In the cohort, 75 were diagnosed with stroke (age 39 to 95 years, median 69 years; 41% female) and 75 were stroke-negative (26 to 91 years, median 66 years; 56% female) based on acute neurological symptoms and imaging findings. Ethics committee approved this retrospective study, and patients’ informed consent was waived. All patients were imaged on a SOMATOM Definition Edge (Siemens Healthineers, Erlangen, Germany) 128-slice CT scanner using a CT stroke protocol previously described in [17]. The methods described below were based on the head portion of the routinely acquired single-phase CTA image volume. The original source images had 512 × 512 matrix size; the pixel resolution varied from 0.32 × 0.32 to 0.58 × 0.58 mm/px with 0.5 mm spacing between slices and 16-bit unsigned integer data type. Head portion of the volume was defined as the region starting from the most superior slice where the skull was present and extending to the slice located 175 mm in the inferior direction.

**Fig. 1:**
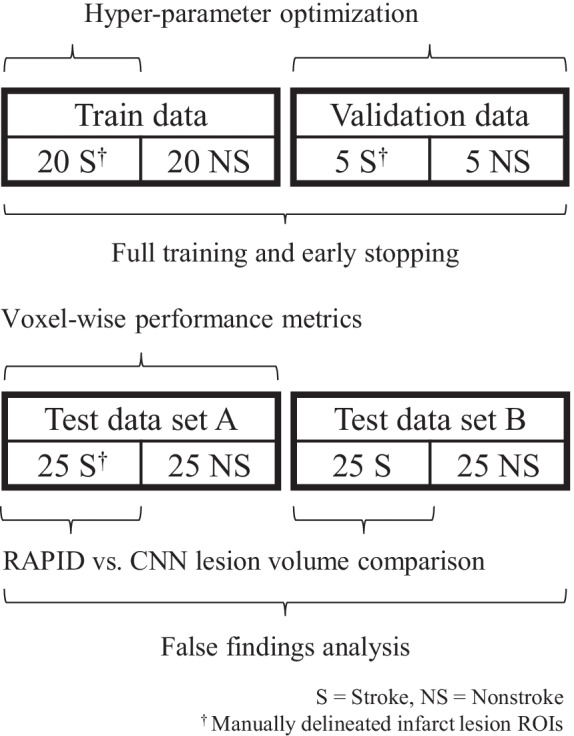
A total of 150 subjects (75 stroke and 75 nonstroke) were divided into four data sets: train, validation, and test sets A and B. Test set A was used in reporting voxel-wise performance relative to the manually drawn lesions. Both test data sets were used for false findings assessment and estimating reliable lesion detection size. The stroke-positives in the test sets were used in comparing the CNN results to the RAPID analysis

The patients were divided into four groups: train, validation, test set A, and test set B (Fig. 1). Train data consisted of 20 stroke and 20 nonstroke cases. Validation data consisted of five stroke and five nonstroke cases. Both test data sets (set A and set B) consisted of 25 stroke and 25 nonstroke cases. High-quality manual segmentations of ischemic changes visible in the CTA were considered the ground truth for CNN training. The CTP study results were available during segmentation. Lesions were manually drawn for the stroke-positive train, validation, and test set A cases. Due to time consuming nature of manual processing, an expansion to the test data (set B) did not include manual lesion delineations. RAPID analysis results were recorded for the stroke cases in the test sets A and B. Test set A was used in evaluating the CNN’s voxel-wise performance against expert lesion delineations. The full (A and B) test set was used for estimating the network’s stroke/no stroke detection accuracy. Manual lesion delineations were made on 3D Slicer image processing platform version 4.8.1 [21] in concurrence by a senior neuroradiologist with over 20 years and a radiologist with 7 years of experience.

### Data Processing

A CNN model for ischemic stroke lesion segmentation was developed. A CTA image volume input was preprocessed prior to feeding into the network. First, the head region was extracted from the volume and background air was excluded from the training and performance evaluations. The volumes were resampled to uniform 0.5 × 0.5 × 0.5 mm/px resolution. The image pixel values were shifted and rescaled so that the 30 to 60 HU range was normalized to − 1 to 1. A secondary image volume was created by left–right flipping a copy of the original image. The flipped and original image volumes were first aligned by a coarse rigid co-registration followed by a non-rigid registration using elastix toolbox version 4.9.0 [22, 23]. The resulting two volumes, i.e., the original and the “flipped and co-registered,” were the two CNN input channels. This guided the network to utilize image information from the matching contralateral side for healthy tissue/lesion classification. The main parameters of the non-rigid B-Spline transform–based registration were as follows: adaptive stochastic gradient descent optimizer with Mattes mutual information metric, four resolution levels, 2048 spatial samples, 16-mm grid spacing, maximum number of iterations 500, and 32 histogram bins. The non-rigid registration allowed more accurate matching of the cortical regions than simple rigid registration by properly aligning the (possibly asymmetric) cranial walls between the image volumes. Image operations such as type conversions, flipping, and resampling were done using Convert 3D (part of ITK-SNAP toolkit) version 1.1.0 [24]. The co-registered image volumes were split into matching sub-volume pairs which were fed into the CNNs during training or inference.

**Fig. 2:**
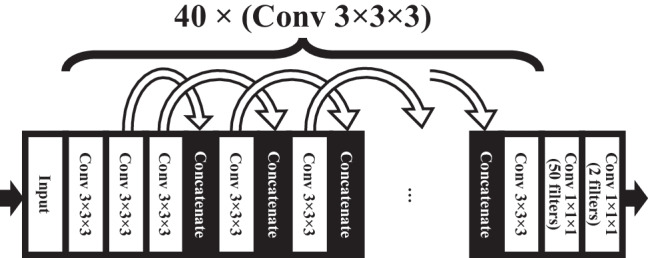
The final patch-based neural network architecture with 3D convolutional layers (Conv) and skip connections. Input during training: preprocessed two-channel 107 × 107 × 107 voxel sub-volumes with mini-batch size eight. Conv 3 × 3 × 3: kernel size 3 × 3 × 3, 16 filters, valid padding, and exponential linear unit activation; curved arrows: skip connection with cropping by one voxel on each side; concatenate: concatenating the outputs of the two preceding layers’ outputs; Conv 1 × 1 × 1 (50 filters): 1 × 1 × 1 convolution with 50 filters and rectified linear unit activation; Conv 1 × 1 × 1 (2 filters): the output layer with two filters (background and lesion) and softmax activation. Due to valid padding, the output dimensions for each individual patch were 26 × 26 × 26 voxels. When performing inference, the patches were stitched together to form a seamless lesion prediction map the size of the input CTA image volume. The total number of trainable parameters was 528,000

A CNN was trained in two phases. First, the architecture was decided based upon validation performance, followed by full training until validation loss stopped improving. The CNN hyper-parameter optimization consisted of selecting the network depth and width, i.e., the number of layers and the number of filters used in the convolutional layers. The search was performed by training eight neural networks on a partial training data set A, i.e., with nonstrokes omitted to save computation time. The networks comprised of C (C = 10, 20, 30, or 40) convolutional layers with F (F = 8, or 16) filters in each layer, followed by one fully connected (i.e., 1 × 1 × 1 convolutional) layer with 50 filters, and a fully connected two-output layer with softmax activation. Skip connections with concatenations were added over most of the convolutional layers to assist gradient backpropagation (see details in Fig. 2). Training was halted at the epoch where validation loss plateaued. The lowest validation loss was seen with the deepest and widest network that was tested (C = 40, F = 16). This network was therefore selected as the basis for the rest of the study. This final 42-layer network architecture was then retrained using the full training data set of 20 stroke and 20 nonstroke cases for 30 epochs after which no further improvement was seen in the validation loss. The networks were implemented in Python 3.5 with Keras library version 2.2.4 [25] on top of Tensorflow version 1.12.0 [26]. Adam optimizer with default parameters with learning rate of 10^−4^ was used with cross-entropy loss function. The class imbalance was mitigated by including equal number of stroke-lesion-positive and stroke-lesion-negative sub-volumes in each mini-batch. In the final training dataset, after air regions were excluded, approximately 3000 positive (lesion) and 71,000 negative (no lesion) patches were present. An epoch was considered when all the positive samples were seen once with a matched number of negative patches. These translated to each epoch consisting of 750 iterations with four positive and four negative patches per minibatch.

**Fig. 3:**
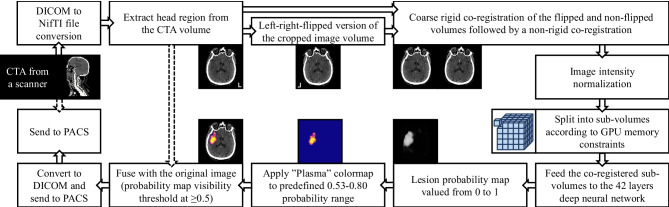
The processing workflow takes approximately 7 min from beginning to receive the DICOM files to completion. A secondary image volume is created from the original CTA where subject’s left and right sides are flipped. This allows the neural network to learn features with information from the contralateral side. The volumes are then co-registered, normalized, split, and fed into the neural network, and the results are sent to PACS

A proof-of-concept automatic image processing pipeline was developed [27]. The system receives a CTA series from a scanner or PACS into DICOM image storage (DCMTK; OFFIS, Berlin, Germany). The pipeline waits the completion of the image transfer, verifies images according to DICOM header rules (Python pydicom library; https://pydicom.github.io), and ensures that the received series form a valid 3D volume. Then, it adds the CTA series into a processing queue database (SQLite database accessed using SQLAlchemy library; https://www.sqlalchemy.org). In the processing steps, the images are converted into NifTI file format (SimpleITK; https://simpleitk.org) followed by pre-processing, neural network inference, post-processing, and conversion back into secondary capture DICOM files. These are then transferred into PACS or a workstation. The general overview of the pipeline and the processing steps are shown in Fig. 3.

The CNN model produces classification confidence estimates (probabilities) for each voxel. A color map called “Plasma” by Smith and van der Walt [28] was chosen for visualizing the lesion probabilities. The map is perceptually uniform, and fairly intuitive, starting from dark blue and ending in bright yellow. It avoids the black–gray–white color spectrum of conventional CT images and is therefore well-suited for image fusion. Predicted values above 0.5 are visible, but the dynamic range in the mid-region of the remaining probabilities is enhanced: the color map range was defined by taking 0.05 and 0.95 quantiles of the predicted probabilities (with lower-bound cut-off ≥ 0.5) in the validation data lesion ROIs.

### Performance Evaluation

Voxel-wise precision, sensitivity, and Sørensen–Dice coefficient between the predicted and manually drawn lesions were calculated for test set A. Due to high class imbalance (the total number of no-lesion voxels outnumbered the lesion voxels sixty to one), measures such as accuracy and specificity were undescriptive of the true performance. Predicted lesion volumes were calculated using a probability threshold of ≥ 0.5 and excluding continuous clusters smaller than 3 mL (per RAPID’s convention). We also investigated at what CNN-predicted lesion volume the presence of a stroke could be reliably identified using the full A + B test sets. The volume threshold for stroke detection was varied and area under the receiver operating characteristic curve (ROC AUC) was calculated. The top-left point on the ROC curve was considered the optimal operating point for stroke detection. Confidence intervals (CIs) were calculated using a bootstrapping method, where resampling with replacement was repeated 10^5^ times while maintaining an equal number of strokes and nonstrokes in each sample.

Linear regression was calculated between the CNN estimated volumes and five RAPID-reported volumes: *T*_max_ > 4 s, *T*_max_ > 6 s, *T*_max_ > 8 s, *T*_max_ > 10 s and ischemic core. Pearson correlation coefficients (*r*) with bootstrap CIs were used to decide which RAPID time point matched the CNN volume estimates the best. Similarly, average volume differences with CIs were estimated by linear regression with forced intersection at the origin. The calculations were performed using MATLAB version 2018b (MathWorks, Natick, MA, USA).

Each of the CNN predictions in the full test set was evaluated visually: the localization of the lesion in the correct vascular territory (based on CTA and perfusion findings) was verified and false positives and false negatives were recorded.

## Results

### Lesion Detection

**Fig. 4:**
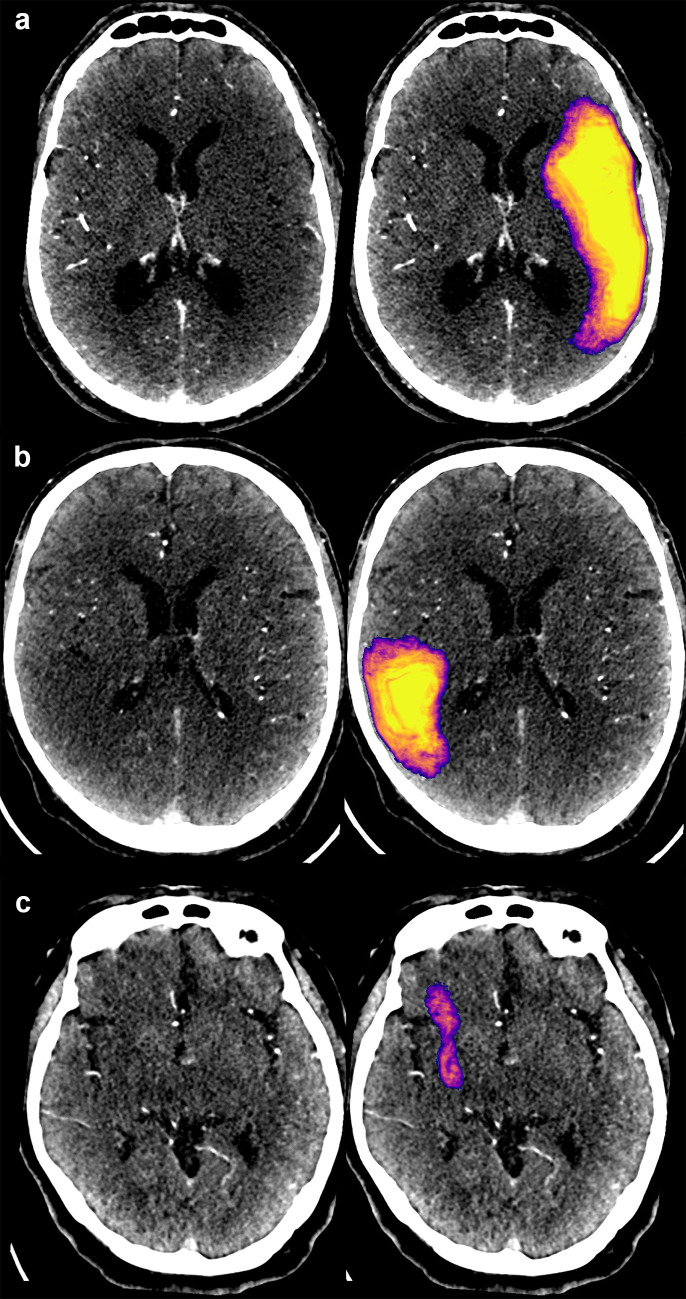
Three examples of ischemic strokes detected by the convolutional neural network (**a**–**c**). In the example **c**, the predicted lesion was smaller than visually estimated by a radiologist

**Fig. 5:**
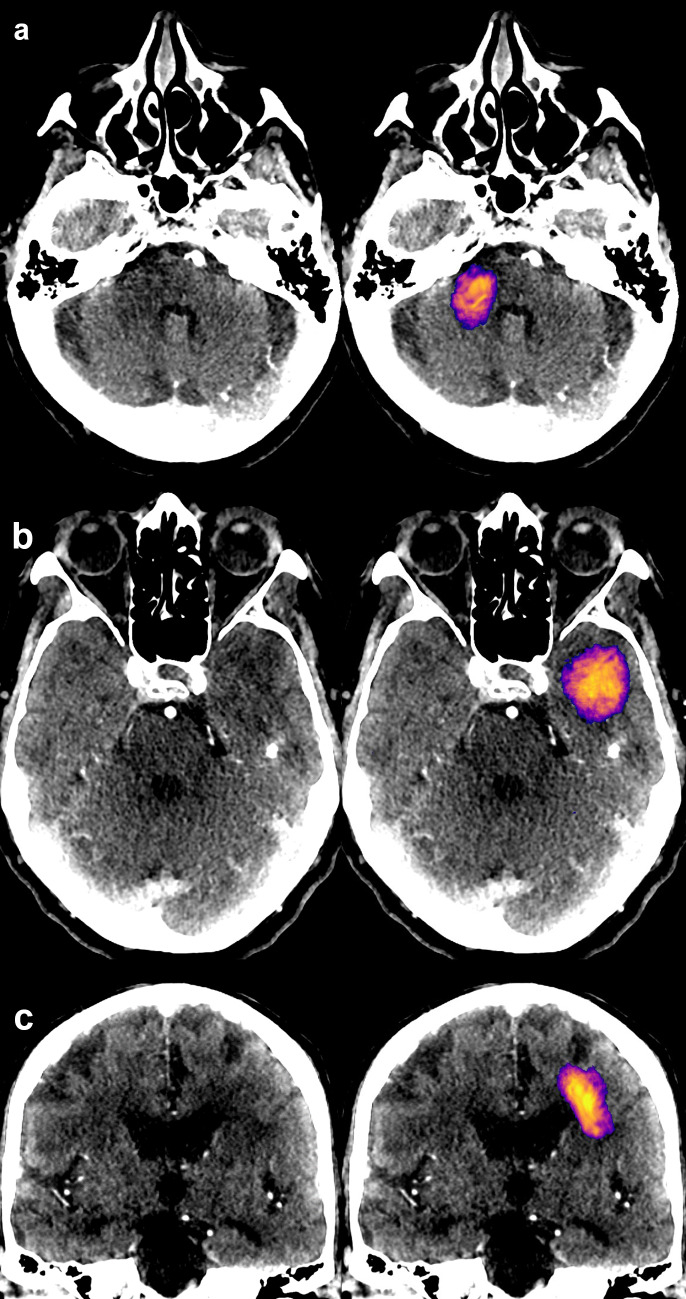
Typical false positives presented **a** in the cerebellum or **b** in the temporal lobe (resulting from beam hardening artifact), or were due to **c** white matter vascular degenerative changes

**Fig. 6:**
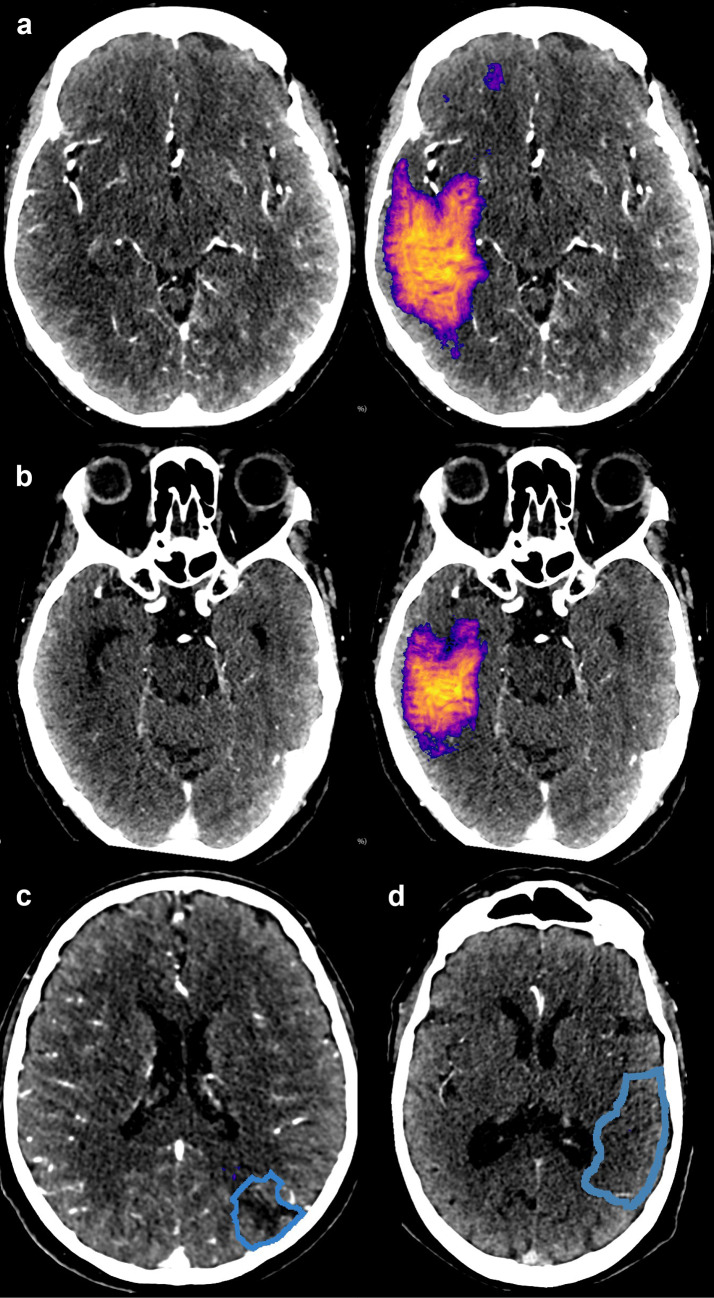
**a**, **b** Two examples of severe (anatomically credible) false positive lesions predicted by the CNN model resulting from hemispheric attenuation asymmetry. In case a, hyper-perfusion on the contra-lateral side was caused by epileptic activity. Two lesions in the test data (blue outline) were completely missed: perhaps due to sulcus-like appearance (**c**) and for an unclear reason (**d**)

Voxel-wise performance results relative to the expert segmentations for test set A were as follows: precision 0.69 (95% CI: 0.60–0.76), sensitivity 0.54 (0.44–0.63), and Sørensen–Dice coefficient 0.61 (0.52–0.67). The predicted volumes were on average 23% smaller than the manual delineations. (See Fig. 4 for examples of correctly identified lesions and Fig. 5 for typical false positives. See Fig. 6 for two severe false positives examples (a and b) and for the two stroke lesions in the test set that were missed completely (c and d). Full processing time, including preprocessing, inference, and network transfers, was 7 min.

**Fig. 7:**
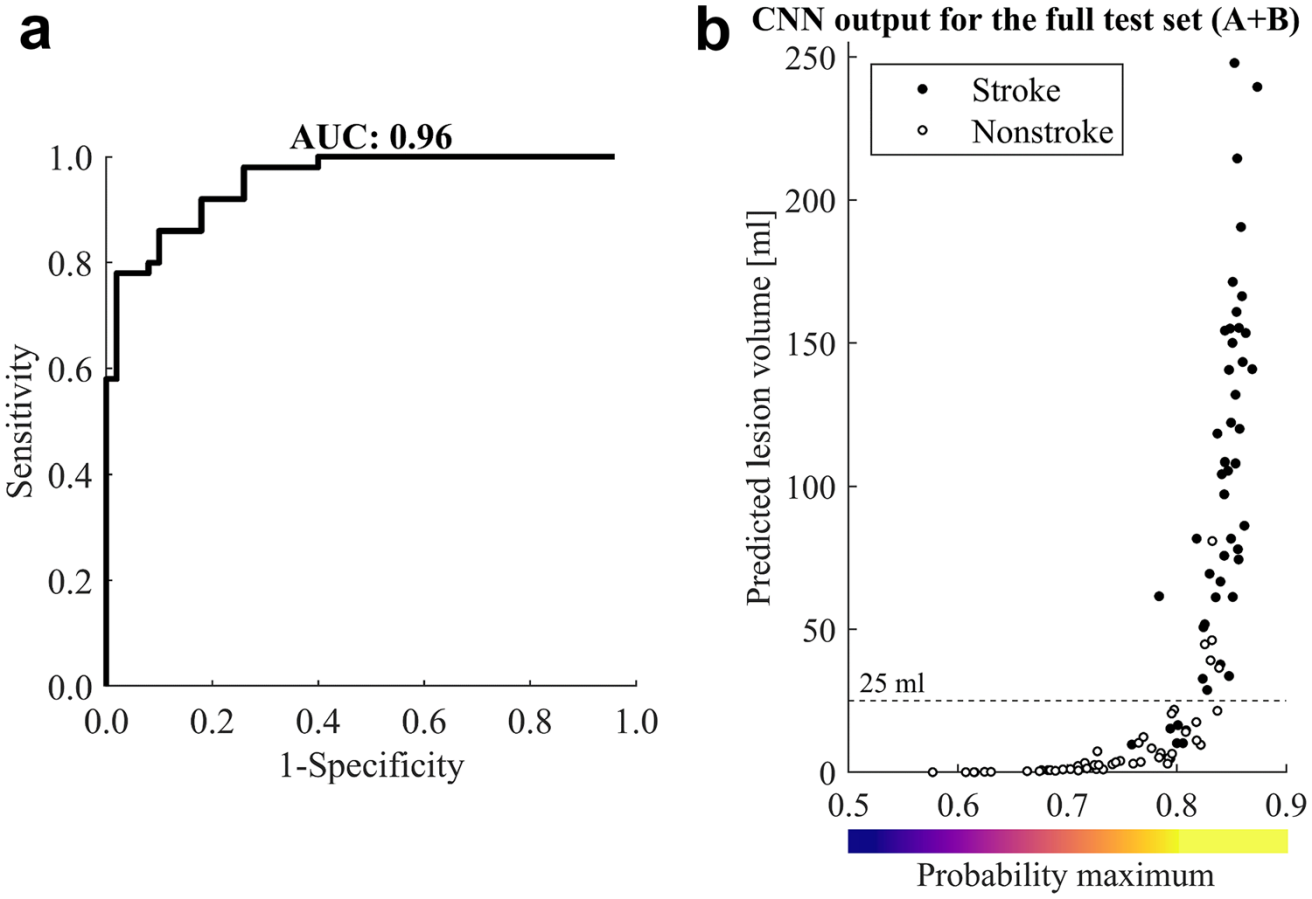
The largest connected lesion volumes were recorded. ROC curve (**a**) was produced by varying volume threshold and comparing against the original stroke diagnosis (black and white dot for stroke and nonstroke). Using a fixed 25-mL limit and predicting that volumes larger than this are present only in stroke patients, 43 true positives, 5 false positives, 45 true negatives, and 7 false negative cases in the test were detected (**b**)

Detected stroke lesion volume could be used to aid in treatment decisions (Fig. 7). Volume cut-off for lesion detection was varied on the full A + B test set (with ground truth being the original stroke diagnosis) producing a ROC AUC of 0.96 (95% CI: 0.92–0.98). The optimal volume threshold maximizing the combined sensitivity and specificity, i.e., corresponding to the closest point to the top-left corner on the ROC curve, was 25 mL. With this value as a decision threshold, 43 true positives, five false positives, 45 true negatives, and seven false negatives were observed, resulting in an accuracy of 0.88 (95% CI: 0.81–0.94). The two completely missed positive cases had erroneous volumes of 9 and 10 mL, i.e., wrong location and below the cut-off. The other five false negatives (based only on the volume threshold) were correctly localized but were underestimated in size. Two of the five false positives resembled true findings whereas the rest could have been ruled out based on other imaging features: not extending to the cortex or an improbable anatomical location.

### Visually Assessed False Positives and Negatives

The CNN-predicted stroke lesions (above 3 mL) were reviewed visually: correct localization was verified and errors were recorded. Connected regions smaller than 3 mL were ignored. No false positives or negatives of any kind were seen on 54 of the 100 test cases (A + B). We identified a total of 67 false findings with 15 cases having more than one type. Erroneous findings were considered severe for nine patients: in seven nonstroke cases, the CNN predicted an anatomically credible “pseudo-lesion” (for examples see Fig. 6a, b), and on two positive cases, the lesion was missed completely (Fig. 6c, d). We speculate that one of the false negatives resulted from sulcus-like appearance (Fig. 6c). No clear reason was found for the other case (Fig. 6d). As a drawback for utilizing hemispheric asymmetry, one large (81 mL) “lesion-mimic” was present due to considerable hyper-attenuation on the contralateral side resulting from epileptic activity (Fig. 6a). The rest of the false findings were considered less severe: either they were anatomically implausible, clearly artefactuous due to beam hardening or resulting from vascular or cortical atrophy (or a combination of these). The most common cause for these “benign” false positives was beam hardening either in the temporal lobe (19) or cerebellum (14). In 14 cases, scattered false positive lesions were seen due to atrophy and subsequent attenuation asymmetry between hemispheres. In 10 instances, the false positive finding was inside a sulcus or otherwise located extra-parenchymally. One old stroke lesion was detected, which was also considered a false positive. In addition, one of the true positives was delineated both considerably smaller than visually estimated (8.1 vs. 31.6 mL) and appeared non-continuous. The lesion would have been better depicted with a lower probability threshold than the predefined ≥ 0.5, but the total number of false positives would have correspondingly increased. The subject in question was the youngest stroke case in the test sets (47 years) which may indicate age dependence in the developed model.

### CT Perfusion Comparison

**Fig. 8:**
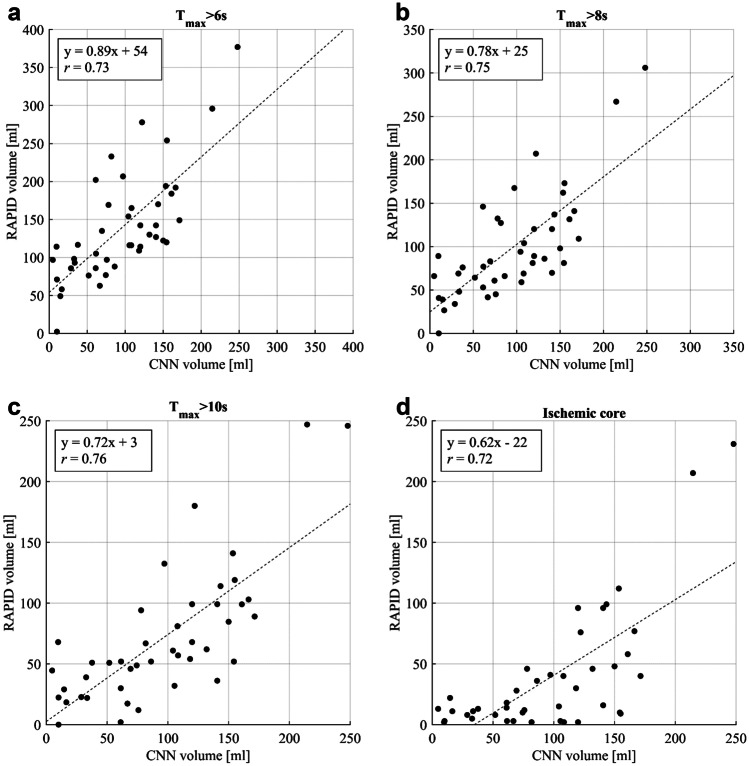
A moderately strong correlation (Pearson’s *r* ≥ 0.72) was seen between the volumes defined by the proposed CTA-based convolutional neural network (CNN) and CTP RAPID analysis volumes (**a-d**: *T*_max_ > 6 s, 8 s, 10 s, and ischemic core) except for *T*_max_ > 4 s (*r* = 0.41, not shown). Linear regression lines are indicated by dashed lines. The highest correlation was for *T*_max_ > 10 s and the best absolute match was for *T*_max_ > 8 s

The largest continuous lesion volumes predicted by the CNN model were recorded and compared to the five RAPID-reported volumes for 43 of the 50 stroke-positives in the test data. Three cases were omitted because no RAPID report was available at the time of analysis. Additional four were excluded because RAPID failed to provide realistic volumes: studies were considered undiagnostic if hypoperfusion (defined as *T*_max_ > 6 s or ischemic core) was reported to both hemispheres and to the posterior fossa. RAPID did not report any problems with perfusion data in any of these cases. Correlations (Pearson’s *r*) between the CNN and RAPID predicted volumes were the following: *T*_max_ > 4 s: *r* = 0.41 (95% CI: 0.14–0.71), *T*_max_ > 6 s: *r* = 0.73 (0.52–0.85), *T*_max_ > 8 s: *r* = 0.75 (0.55–0.86), *T*_max_ > 10 s: *r* = 0.76 (0.58–0.86), and ischemic core: *r* = 0.72 (0.52–0.84) (Fig. 8). On average the corresponding relative volume differences between RAPID and CNN were (positive meaning RAPID produced larger volumes): + 101% (95% CI: + 79 to + 133%), + 31% (+ 17 to + 45%), − 3% (− 16 to + 9%), − 26% (− 40 to − 15%), − 54% (− 71 to − 40%), respectively. The highest correlation (“moderately strong” per convention in [29] ) was seen for RAPID *T*_max_ > 10 s volume, although only *T*_max_ > 4 s correlation differed significantly from the others. The most accurate absolute match between the CNN- and RAPID-reported volumes was for *T*_max_ > 8 s.

## Discussion

In this study, a fully automated, patch-based 3D convolutional neural network model was applied to detect AIS lesions, and 91% stroke detection accuracy for the study population was achieved. The robust preprocessing consisted of only resampling, pixel value scaling, and registering the two hemispheres: no additional ad hoc steps such as histogram equalization [8], atlas-based template matching [30], skull stripping [14], or separation of tissue types [15] were needed, and no cases were excluded due to improper registration [13]. Also, no morphological or other post-processing, except for predefined > 3 ml threshold, was required. The network demonstrated an affinity for certain false positive inducing imaging features, which were primarily below 25 mL in volume. This exploratory study concerned only patients with suspected MCA strokes, and therefore the model’s applicability to other territories, such as posterior circulation strokes, can be expected to be very limited.

Two other groups (Sheth et al. [15] and Hilbert et al. [13] ) have also demonstrated the applicability of CNNs and CTA in ischemic stroke detection and neither applied strict high-quality manual lesion segmentations in their studies, but rather opted for more big data approach (297 cases and 1301 cases for training, respectively, vs. 50 cases in this study). The volumetric comparison to RAPID suggested that the proposed model produces ischemic lesion estimates that correspond approximately between traditionally considered penumbra (*T*_max_ > 6 s) and the ischemic core. Notably, CNN volume estimates were categorically larger than RAPID ischemic core estimates. Directly estimating ischemic core volume may also be possible from CTA alone as demonstrated by Sheth et al. They reported “an acceptable correlation” between RAPID ischemic core volumes and network output probabilities (*r* = 0.70). In the current study, a similar correlation between the RAPID core volumes and the CNN-predicted volumes was observed (*r* = 0.72). It is notable that neither of the networks was trained explicitly on exact ischemic core segmentations or exact ischemic core volumes.

**Fig. 9:**
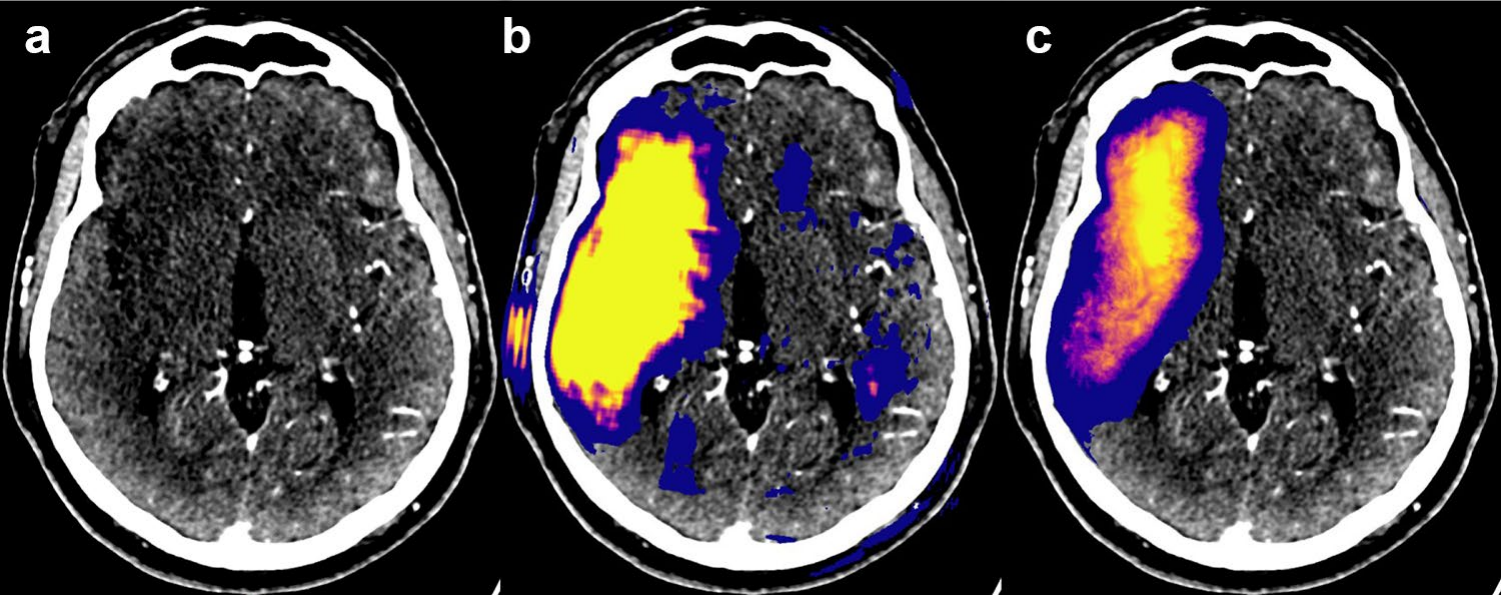
A similar performance was seen between the models from the previous [17] and the current study. In the example case (**a**), the old model (**b**) produced less conservative lesion estimation and was more prone to false positives than the current model (**c**). The probability threshold in the images **b** and **c** is set to ≥ 0.05 (values below 0.53 are blue). The most striking visual differences between the predictions of the two CNNs were the greater range of probability values and less rugged edges produced by the current model

The feasibility of ischemic stroke detection using a CNN with CTA image inputs was confirmed in a preceding study [17] in which a dual-pathway CNN architecture DeepMedic [19] was used. In the current study, the methodology was improved in three primary ways: (1) time spent on manual delineations for the training set was increased aiming to reduce training data noise, (2) a new CNN model with different network architecture was trained, and (3) the inference was fully automated. Because the networks in the previous and the current study were trained on different data sets, no extensive comparison was made; however, a brief discussion is provided in the following. Based on comparison during network development, the CNN models of the previous [17] and the current study provided similar voxel-wise performances and lesion detectability (for a visual comparison see Fig. 9). However, anecdotally, the new network produced more localized predictions and was less prone to false findings and the overall performance was more consistent. It also tended to be more conservative in lesion segmentation lowering voxel-wise evaluated performance. Also, in the primary testing, it was observed that networks with multi-resolution approaches (using pooling and upsampling layers) tended to produce certain repeated-edge-like artifacts near bony cranial walls. These were also visible in the previous study’s network predictions and presented both intra- and extra-cranially. (For an extra-cranial example see the left-hand side of the Fig. 9b). These were probably due to network memorization effects (following likely from limited training data) where mere closeness to the skull tended to be indicative of a stroke lesion extending to the cortical surface. These artifacts were not seen in the predictions produced by the proposed network which was the main motivation for the current single resolution-level architecture choice. Memorization was also mitigated by maintaining a relatively low number of trainable parameters.

The final CNN had only half-a-million trainable parameters without pooling, dropout, or normalization layers. The difference is quite drastic when compared, for example, to a high-performing ImageNet classification network by Mahajan et al. with up to 829 million parameters [31]. A huge network would most likely require a larger training set to avoid overfitting. The large number of voxels and large anatomical variation combined with highly subjective nature of exactly delineating the lesion borders (i.e., training data noise) could result in superfluous correlations when using networks with strong representative power and larger receptive fields. The latter could, for example, mean zealously excluding regions never seen in the training data only based on the surrounding anatomy rather than actual information content at the specific region. Although 40 consecutive 3 × 3 × 3 convolutional layers could in principle have 81-voxel-wide receptive field, it has been postulated that in fully convolutional hierarchies the effective receptive fields tend to be only a fraction of the theoretical ones [32]. Therefore, the non-pooling architecture selected for this study possesses a (perhaps desirable) handicap for extinguishing false positives on surrounding anatomy alone. The authors postulate that the network learned a local and, to some extent, explainable extraction of information, even if it suffers from non-stroke-related attenuation asymmetries. The decision is primarily driven by a combination of local texture and differences in iodine-uptake and brain tissue densities between the hemispheres. These features can be, when necessary, interpreted visually from the original images, and the possible true or false findings can be put in a correct physiological or anatomical context.

There are several approaches to counter the aforementioned pitfalls. The effect of ground truth noise could be mitigated by relaxing the loss function penalty for near-exact match: for example, Lisowska et al. used a “do not care” zone at the manual vessel segmentation edges [11]. The amount of training data in this study was mainly limited by the arduousness of manual segmentation. Deep learning allows different tactics to circumvent this cost: Hilbert et al. used treatment outcome [13], Barman et al. used stroke/nonstroke [30], and Sheth et al. used the presence/absence of large vessel occlusion and dichotomized RAPID-reported ischemic core volumes as the training targets [15]. This way it was possible to substantially increase the number of training samples. However, in these studies, to interpret from which regions the network derives its decisions, activation maps needed to be approximated. This is not necessary for the segmentation approach. The exact localization of the positive and negative findings allows more straightforward method development. Secondly, the model in the current study provides exact volume estimates whereas Sheth et al. limited the core volume investigation to clinically defined values of ≤ 30 mL and ≤ 50 mL. In this study, RAPID values were used as a semi-external validation method (same session but different scan type and algorithm) instead of being a basis for training. Lastly, our method was prone to specific image artifacts and physiological changes which could be mitigated by a more advanced CNN model but also by technical improvements in image acquisition. Considering emerging technologies, along with improved image reconstruction and artifact reduction, it has been reported that dual energy CT could aid in blood/parenchyma/bone distinction and reduce artifacts [33].

In the current study, memory and computation hungry 3D convolution filters and high-resolution input images were used, which limits the versatility of different network architectures that can realistically be adopted. Inference speed is also affected by the large number of convolutional layers using valid padding and subsequent need to process the image in a large number of overlapping sub-volumes with redundant computations. The time requirements for both training and inference were the reason why the number of layers and filters was not further increased during the hyper-parameter search. Hilbert et al. drastically reduced the dimensions by maximum intensity projection over the whole skull-stripped image volume to single axial image [13]. A plethora of other options include simply using 2D slices as inputs, some version of 2.5D (e.g., tri-planar) approach [34], or multi-scale processing either for the inputs [10] or inside the network [12]. These are compromises between available information and computational cost, and the current choice of fully 3D CNN aims to maintain all of the local information regardless of the chosen reconstruction orientation. This is analogous to using several millimeter slice thicknesses in multi-planar CT reading, but without a priori decision how to combine the neighboring voxels. The low contrast-to-noise ratio in CT is both due to small differences in x-ray attenuation in soft tissues (and low concentration iodine) and the desire to keep radiation dose levels as low as possible following ALARA principle. Non-contrast CT alongside with CTA could be utilized in better extracting the presence of contrast-agent. Multi-modal approaches where several image volumes are fed into a neural network are more often used in MR research [9, 35, 36] than in CT. However, in the previous study, the inclusion of non-contrast CT did not improve the performance [17], and the unimodal approach was therefore selected for this study.

The proposed CNN lesion volume estimates were somewhere between RAPID predicted *T*_max_ > 6 s volume, which is considered the best indicator of ischemic penumbra [37], and the ischemic core volume. There was no diffusion-weighted (considered golden standard) or follow-up imaging analysis to test the accuracies of these methods. It has been postulated that current CTA with fast acquisition times is more heavily cerebral blood flow than volume weighted, i.e., possibly overestimating ischemic core volumes compared to CTP, which may in part explain the results [38].

Study limitations include dataset size, possible selection biases from the inclusion criteria (especially focusing only on the suspected MCA strokes), both training and test data being from a single scanner (unknown generalizability), and the subjectivity resulting from difficulty of visually discerning abnormal regions in the CTA images. The sample size of 50 infarct positive cases was relatively small and weighted on larger sized infarcts. Both CTP and CTA may suffer from various factors detrimental to image quality, such as abnormal circulation or patient movement and neither method successfully detected all stroke cases. The inclusion criteria in this study required that the study included a CTA series with good image quality, and no conclusions can be drawn for performance on poor-quality images. Assessing the potential impact on the patient care and determining how CTP and CTA analyses complement each other warrant a further study, preferably including follow-ups and a wide range of stroke sizes and sites, and with careful analysis of both algorithms’ success rates and failure reasons in complete clinical populations.

Current guidelines regarding treatment selection in AIS are based on large trials that investigated the safety and efficacy of thrombolysis and mechanical thrombectomy in an extended time window: 6–16(–24) hours for thrombectomy and 4.5–9 h for thrombolysis [39–41]. Patients for these studies were selected based on a mismatch between ischemic core and penumbra and the detected ischemic core was to be < 21–70 mL depending on other factors such as the age and functional capacity of the patient. The optimal volume threshold maximizing sensitivity and specificity for the proposed CNN model was 25 mL. To put the value in context, the average volume of the whole MCA territory is 284 mL and the average volume of the basal ganglia is 10 mL [42]. The effect is also seen in Fig. 8d where there is visibly very little correlation between the two methods for small RAPID-reported core volumes. Although the CNN model did not allow for exact ischemic core and penumbra mismatch estimation from CTA, the results suggest that CNN powered CTA could offer supporting information for patient diagnosis or triage in hospitals where CTP is not available. These findings are in line with conclusions by Hilbert et al. [13] and Sheth et al. [15]. Apart from extending the search for optimal hyper-parameters, there are several approaches to further develop the proposed methods. For example, a secondary CNN at lower resolution could be used to rank out anatomically improbable false positives (especially if targeting individual vessels); the models could be trained based on control MR or CT images, or on the CTP RAPID–derived core and penumbra estimates.

## Conclusions

A fully automated deep CNN model can be trained on a relatively small training set to accurately detect acute ischemic stroke lesions from CTA images with a correlation to RAPID-reported CTP volumes. The results could be further improved by extended network optimization and a training set with larger variety of normal tissue and lesion manifestations. The total processing time for a single CTA volume was 7 min.

## Data Availability

Data supporting the study findings are obtainable from the corresponding author upon reasonable request.
